# Antinociceptive Effect of Liposomal Bupivacaine Formulations After Intrathecal Administration in Rats

**DOI:** 10.3906/sag-1806-200

**Published:** 2019-02-11

**Authors:** İlker YİĞİT, Berrin GÜNAYDIN, Hakan O. EMMEZ, Orhan ULUDAĞ, Nur Banu BAL, Tuncer DEĞİM

**Affiliations:** 1 Department of Anesthesiology, Faculty of Medicine, Gazi University, Ankara Turkey; 2 Department of Neurosurgery, Faculty of Medicine, Gazi University, Ankara Turkey; 3 Department of Pharmacology, Faculty of Pharmacy, Gazi University, Ankara Turkey; 4 Department of Pharmaceutical Technology, Faculty of Pharmacy, Gazi University, Ankara Turkey

**Keywords:** Extended release formulation, liposomal bupivacaine, intrathecal, rat

## Abstract

**Background/aim:**

Based on our previous in vitro study with multilamellar liposomal bupivacaine (MLB) versus bupivacaine alone in artificial cerebrospinal fluid, we aimed to investigate in vivo antinociceptive effect of intrathecal MLB by determining tail flick latency (TFL) time after thermal stimulation in rats.

**Materials and methods:**

After preparing MLB and high-yield drug entrapment in liposome (HYDEL) bupivacaine, 18 female Wistar rats were assigned to 3 groups as control (bupivacaine) and study groups (MLB and HYDEL bupivacaine) including 6 rats in each group to administer these drugs intrathecally. Antinociceptive activity was determined in terms of TFL time after thermal stimulation. Maximum possible effect (MPE) calculated from TFL times and rats with motor block were documented.

**Results:**

TFL times after intrathecal injection of HYDEL bupivacaine were significantly longer than that of the control and MLB groups (P < 0.05) and returned to baseline 180 min after intrathecal injection. MPE (100%) with intrathecal HYDEL bupivacaine occurred between 10 to 45 min. Afterwards, MPEs were 70% and 50% for the control and MLB groups, respectively. Motor block disappeared after 20 min in the study groups while it lasted 75 min in the control.

**Conclusion:**

Intrathecal administration of MLB and HYDEL bupivacaine in rats resulted in longer duration of antinociceptive activity with shorter motor block duration.

## 1. Introduction

Experimental and clinical studies with long-acting amid-type local anesthetics encapsulated into liposomes have been conducted to provide a prolonged local anesthetic effect, a reduction of the plasma peak drug concentration, and the safe administration of larger doses, which further prolongs the duration of analgesia. All these studies with liposomal local anesthetics have been done by either epidural administration or local infiltration (1,2).

We previously showed the in vitro slow releasing pattern of multilamellar liposomal bupivacaine (MLB) when compared to bupivacaine alone as control in the artificial cerebrospinal fluid (CSF) (3). Based on our previous in vitro study, we hypothesized that an MLB formulation might produce a longer antinociceptive effect associated with differential block as compared to bupivacaine alone. So far, no study has been conducted by injecting liposomal local anesthetics in the intrathecal space. Therefore, we aimed to investigate the possible in vivo antinociceptive effect of intrathecal liposomal bupivacaine by determining tail flick latency (TFL) time after thermal stimulation in rats. 

## 2. Materials and methods

The study was approved by the Institutional Animal Care and Use and Ethics Committee of Gazi University (Research Project Number: G.U.E.T-13.043.29/05/13) and conducted at the Pharmacology and Pharmaceutical Technology Research Laboratories of Faculty of Pharmacy at Gazi University.

### 2.1. Preparation of liposome formulations 

Structurally multilamellar liposomes were prepared from dipalmitoyl phosphatidyl choline (DPPC)-cholesterol in 50% ratio using the dry-film hydration by vortex mixer (Firlabo, Lyon, France) as described by Serikawa et al. (4) and Kajiwara et al.(5) Twenty mg of DPPC (Sigma, St. Louis, MO, USA), 20 mg of cholesterol (Sigma), 50 mg (10 mL) of bupivacaine HCl (Bustesin 0.5% flacon, Vem, İstanbul, Turkey), and 6 mL of methanol (Merck, Germany) were kept ready to use (3). Then, two different formulations were prepared as MLB and high-yield drug entrapment in liposome (HYDEL) Bupivacaine (6,7). 

#### 2.1.1. Preparation of MLB 

**Step 1: **20 mg of DPPC, 20 mg of cholesterol, and 6 mL of methanol were distillated in the rotavapor device (Büchi Rotavapor R-200, Flawil, Switzerland). Solvent containing methanol was removed at 340 mbar pressure where water bath (Nuve BM 402, Ankara, Turkey) temperature was 60 °C and cooler water bath temperature was 10 °C. After adding 10 mL of bupivacaine 0.5% (50 mg), the resulting precipitation was kept for 30 min in the ultrasonic bath (Bandelin Sonorex, Berlin, Germany) to provide a homogeneous distribution.****

#### 2.1.2. Preparation of HYDEL bupivacaine

Step 1 was followed by washing liposomes by centrifugation (Jouan MR 1822, Danfoss, Slovenia) at 10,000 ×*g* for 15 min to prepare HYDEL bupivacaine. Nine mL of the liquid was removed from the tube, while the remaining part was vortexed in the ultrasonic bath for 15 min to achieve homogenous suspension.

### 2.2. Experimental protocol

Eighteen Wistar female rats weighing between 250 and 300 g were randomly assigned to 3 groups as indicated below:

Control group (n = 6): 0.5% bupivacaine HCl,

MLB group (n = 6),

HYDEL bupivacaine group (n = 6). 

The rats were kept ready to administer sevoflurane (Sevorane®, AbbVie, İstanbul, Turkey) soaked gauze that may allow them to awake within approximately 3 min. Then, 30 µL of one of the formulations was injected into the intrathecal space with a sterile disposable h 26 gauge ½ inch hypodermic needle (AYSET, Adana, Turkey) at lumbar region after achieving a positive indication of electrical shock-like tail movement as described (8). 

### 2.3. Assessment of antinociceptive effect 

**Figure 1:**
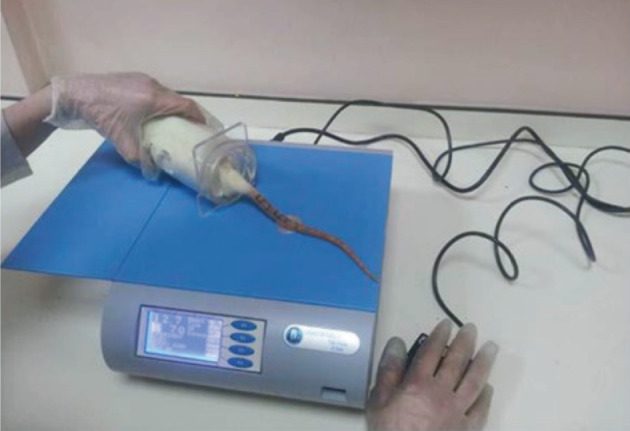
Rat on the tail flick device

Analgesia was assessed by determining TFL time using tail flick device (Figure 1) (9). 

#### 2.3.1. Measuring TFL time 

Thermal stimulation (54 °C) was applied to 3–4 cm proximal part of the tail of the rats placed on the Tail-Flick device (Ugo Basile 37360, Geminio, Italy). Time elapsed from the onset of stimulation to tail flick was defined as TFL time in seconds (s). Cut-off time was accepted as 12 s (9). Mean TFL was found to be approximately 3–4 s and considered the baseline for the present study. Then, baseline TFL (before study onset) and after intrathecal administration of study drugs were recorded at 10, 20, 30, 45, 60, 75, 90, 120, 150, 180, 240, and 300 min.

#### 2.3.2. Maximum possible effect 

The TFL was converted to percent maximum possible effect (MPE) for each group. 

It was calculated dividing baseline TFL by cut off baseline and the result is multiplied by 100%) (9).

_MPE =_ (Baseline TFL) _× 100%_

 (Cut-off baseline)

### 2.4. Assessment of motor function 

Motor function of the lower extremity was assessed by allowing each rat to walk on the table at 10, 20, 30, 45, 60, 75, 90, 120, 150 180, 240, and 300 min and recorded.

Motor block degree was evaluated as complete, partial, or no block at all (10):

· Zero degree (complete block): rats cannot walk/move,

· 1st degree (partial block): rats can hardly walk/move,

· 2nd degree (no motor block at all): rats can move/walk. 

### 2.5. Termination of the study

Animals were sacrificed by intraperitoneal injection of thiopentone of 120 mg kg−1 24 h after the study.

### 2.6. Statistical analysis 

All variables were expressed as mean ± standard error of mean (SEM). After descriptive statistics, repeated measures ANOVA was used within each group. Multiple comparisons between every two groups were made by Student’s t-test.

Data of the study showed normal distribution when analyzed by the computer program GraphPad Prism (version 5.00 for Windows: GraphPad Software, San Diego, CA, USA). A P-value less than 0.05 was considered statistically significant.

## 3. Results 

### 3.1. Antinociceptive effect

#### 3.1.1. The TFL time

**Figure 2:**
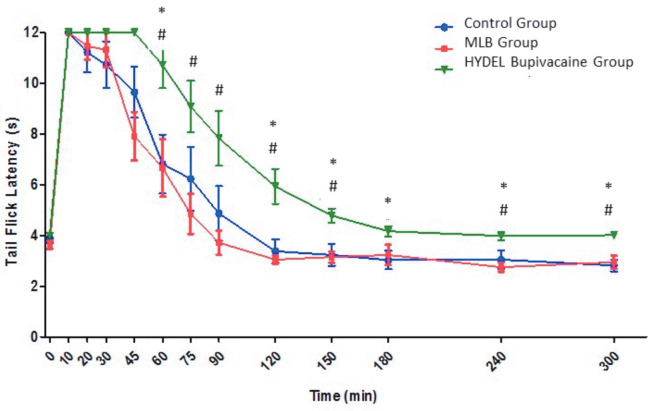
Tail flick latency times after intrathecal administration of MLB and HYDEL bupivacaine versus control in rats (n=6 in each group). HYDEL= High Yield Drug Entrapment in Liposome. *:P<0.05 versus control group #:P<0.05 versus MLB group

The changes in TFL over time in control versus study groups (MLB and HYDEL bupivacaine) were displayed in Figure 2. Predetermined cut-off time (12 s) was achieved in 10 min in all groups. However, it persisted until 45 min only in the HYDEL bupivacaine group.

The TFL times were significantly longer in the HYDEL group when compared to MLB group at all measurement intervals between 60 and 300 min after intrathecal injection. 

Significantly longer TFL times in the HYDEL group vs the control group were observed at 60, 120, 150, 180, 240, and 300 min after intrathecal injection (P < 0.05). 

Approximately 180 min after intrathecal injection, TFL times returned to cut-off time and persisted until the end of 300 min (Figure 2). 

Therefore, TFL times in HYDEL group recorded at 60, 120, and 150 min (11, 6, and 5 s, respectively) were considered significant when compared to both the MLB and control groups (P < 0.05) (Figure 2). 

#### 3.1.2. The MPE 

**Figure 3:**
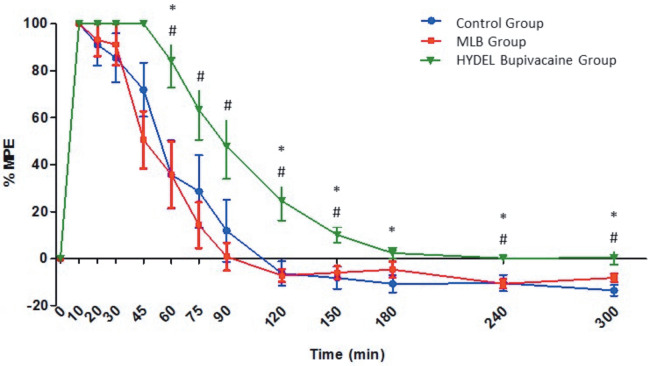
Maximum Possible Effect (MPE) indicated over time after intrathecal administration of MLB and HYDEL bupivacaine versus control in rats (n=6 in each group). *:P<0.05 versus control group #:P<0.05 versus MLB group

The MPEs of the 3 groups over time were displayed in Figure 3. The MPEs were significantly longer in the HYDEL bupivacaine group when compared to the MLB group at all measurement intervals between 60 and 300 min after intrathecal injection (P < 0.05). 

Significantly longer MPE rates in the HYDEL bupivacaine group than those of the control group were observed at 60, 120, 150, 180, 240, and 300 min after intrathecal injection (P < 0.05). Then, MPEs recorded at 240 and 300 min declined to zero. 

### 3.2. Motor function 

**Figure 4:**
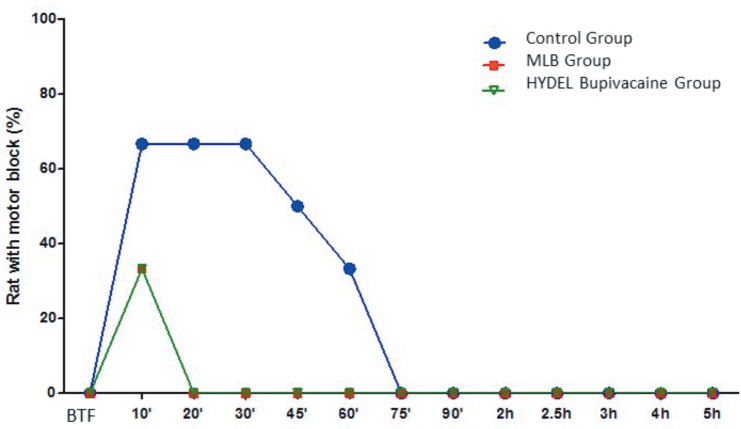
Percentage of rats with complete or partial motor block (zero or 1st degree) over time after intrathecal administration of MLB and HYDEL bupivacaine versus control in rats (n=6 in each group). BTF: Before Tail flick *:P<0.05 versus control group #:P<0.05 versus MLB group

Rats with complete or partial motor block (0 or 1st degree) over time after intrathecal administration are shown in Figure 4. Approximately 70% of rats had complete or partial motor block at 10 min and there was no motor block at 75 min in the control group, whereas approximately 35% of the rats had complete or partial motor block at 10 min and there was no motor block at 20 min in both the MLB and HYDEL groups.

## 4. Discussion

In the current in vivo study, primarily, the antinociceptive effect of MLB versus control after the intrathecal administration was demonstrated in rats. Secondly, HYDEL bupivacaine was found to be a superior formulation than MLB.

Liposomal bupivacaine was used with different routes in the management of postoperative pain relief in experimental and clinical studies. It was effectively used for postoperative analgesia either by local infiltration in humans or extradurally in rabbits (1,2). The first study demonstrated that the distribution of extradural effect of MLB was less than that of plain bupivacaine, while the radioactivity in the lumbar spinal nerves peaked in the first hour and remained higher after 4 h when compared with plain bupivacaine in rabbits (2). Afterwards, liposomal bupivacaine was shown to be effective for treating postoperative pain after burniectomy and hemoroidectomy when used via local infiltration compared to placebo in humans (1).

Hereby, we have demonstrated the in vivo antinociceptive effect after intrathecal administration of either MLB or HYDEL bupivacaine due to their extended release pattern in rats. Antinociceptive effect of HYDEL bupivacaine in terms of TFL time and MPE was superior to MLB. Motor block duration was shorter with both MLB and HYDEL bupivacaine than that of control. The underlying mechanism of faster return of motor function might be the in vivo slow releasing pattern of the liposomal drugs in the intrathecal space.

EXPAREL (bupivacaine in combination with proven product delivery platform, DepoFoam®) is a controlled release formu­lation that is prepared by multivesicular technology. Multivesicular liposomes (MVL) are different from either unilamellar or multilamellar ones. The MVL are larger than the traditional unilamellar (<1 μm) and multilamellar (1–5 μm) liposomes. Depofoam, which is an MVL preparation, has a nonconcentric multiple lipid lay­er, while multilamellar liposomes have concentric lipid bilayers (11–13). The present in vivo investigation is the first experimental study that demonstrated the in vivo extended release pattern of struc­turally MLB after intrathecal administration in rats. We determined the antinociceptive activity by recording TFL time after thermal stimulation in anesthetized rats that received either intrathecal MLB or HYDEL bupivacaine.

In humans, only a single dose of liposomal bupivacaine for wound infiltration to provide postoperative pain relief is FDA-approved (14). In healthy volunteers, the effects of epidural administration of a single dose of DepoFoam formulation revealed a long lasting sensory block without prolongation in motor block (15). 

Long ago, determination of antinociceptive effect of local anesthetics without liposomes after intrathecal administration in rats was represented by MPE (9). Similarly, we used MPE to compare the antinociceptive effects. We observed a significantly higher MPE with HYDEL bupivacaine than those of the MLB and control 60 min after intrathecal use. Additionally, 100% MPE with HYDEL bupivacaine was observed between 10 and 45 min after intrathecal use. Interestingly, simultaneous mean MPEs at 45 min after intrathecal administration were 70% and 50% for the control and MLB groups, respectively. Based on these findings, HYDEL bupivacaine had the greatest antinociceptive effect. We as­sumed that the concentric lipid bilayer of the HYDEL bu­pivacaine formulation might have played a more important role in the extended release pattern by limiting the drug release to a much greater extent than MLB.

In studies with local anesthetics only (without liposomes), 100% MPEs obtained from either 0.5% bupivacaine or 0.5% levobupivacaine lasted half an hour (9), whereas 90% MPE of HYDEL bupivacaine lasted 300 min (5 h) and then MPE became 0%. Therefore, we discontinued data collection and recording after 5 h. Additionally, 70% of the rats in the control group and 30 % rats in the study groups had complete or partial motor block at 10 min. Motor block disappeared at 75 min and 20 min in the control and study (MLB and HYDEL Bupivacaine) groups, respectively.

We previously showed that bupivacaine was released from all liposomal bupivacaine formulations in vitro (3). The release rates were slower depending on liposomal formulations, which might be be­cause of the controlled release of active substance by the li­posome’s lipid bilayers. When liposomes were separated and redispersed, the drug content decreased. Therefore, the total released drug was found to be low. However, when the outer medium also contained the study drug, total release was found to be high. In all formulations, the lipid wall of liposome limited the drug release, which revealed that the lipid bilayer could have played an important role (3).

Despite the concerns, injection of pharmacologically active liposomal bupivacaine into brachial plexus did not result in neurotoxicity (16). When rabbits were assigned into 5 groups (n = 6 in each) to be injected intrathecally 0.3 mL of NaCl solution (control), 2% tetracaine, 10% lidocaine, 2% bupivacaine, or 2% ropivacaine, the sensory and motor functions in the lidocaine group were significantly worse than in the other groups. The extent of characteristic histopathologic vacuolation of the dorsal funiculus and chromatolytic damage of motor neurons was lidocaine = tetracaine > bupivacaine > ropivacaine (17). 

These results may be promising, if intrathecal extended release profiles are reproduced successfully by using MLB.

Recently, the potential role of liposomal bupivacaine as an extended release bupivacaine has been reviewed not only in chronic pain states but also in epidural and intrathecal practice (15,18–20). In healthy volunteers, epidural administration of liposomal bupivacaine provided longer duration of sensory block with shorter duration of motor block than nonliposomal bupivacaine and intradermal injection of multivesicular form produced a prolonged duration of analgesia in a dose-dependent manner (15,19). Another study with MLB in rats showed that there was an 8-fold increase versus placebo in the duration of wound analgesia (20). To the best of our knowledge, no prior comparative study has been conducted on the intrathecal use of MLB formulations to demonstrate the extended controlled release profile in vivo until now. This is the first study to show in vivo extended antinociceptive efficacy after intrathecal injection in rats.

In conclusion, intrathecal administration of MLB and HYDEL bupivacaine in rats resulted in longer duration of antinociceptive activity with shorter motor block duration. 

Therefore, these results are encouraging for providing prolonged neuraxial analgesia and/or anaesthesia with single injection of these bupivacaine formulations in the future.
